# Segmentation of Spontaneous Intracerebral Hemorrhage on CT With a Region Growing Method Based on Watershed Preprocessing

**DOI:** 10.3389/fneur.2022.865023

**Published:** 2022-03-29

**Authors:** Zhengsong Zhou, Hongli Wan, Haoyu Zhang, Xumiao Chen, Xiaoyu Wang, Shiluo Lili, Tao Zhang

**Affiliations:** ^1^Department of Electronic Information Engineering, Chengdu Jincheng College, Chengdu, China; ^2^Department of Epidemiology and Health Statistics, West China School of Public Health and West China Fourth Hospital, Sichuan University, Chengdu, China; ^3^Department of Neurosurgery, West China Hospital, Sichuan University, Chengdu, China

**Keywords:** ICH, image segmentation, watershed algorithm, region-growing, CT

## Abstract

Intracerebral hemorrhage (ICH) poses a great threat to human life due to its high incidence and poor prognosis. Identification of the bleeding location and quantification of the volume based on CT images are of great significance for assisting the diagnosis and treatment of ICH. In this study, a region-growing algorithm based on watershed preprocessing (RG-WP) was proposed to segment and quantify the hemorrhage. The lowest points yielded by the watershed algorithm were used as seed points for region growing and then hemorrhage was segmented based on the region growing method. At the same time, to integrate the rich experience of clinicians with the algorithm, manual selection of seed points on the basis of watershed segmentation was performed. With the application of segmentation on CT images of 55 patients with ICH, the performance of the RG-WP algorithm was evaluated by comparing it with manual segmentations delineated by professional clinicians as well as the traditional ABC/2 method and the deep learning algorithm U-net. The mean deviation of hemorrhage volume of the RG-WP algorithm from manual segmentation was −0.12 ml (range: −1.05–1.16), while that of the ABC/2 from the manual was 1.05 ml (range: −0.77–9.57). Strong agreement of the algorithm and the manual was confirmed with a high intraclass correlation coefficient (ICC) (0.998, 95% *CI*: 0.997–0.999), which was superior to that of the ABC/2 and the manual (0.972, 95% *CI*: 0.953–0.984). The sensitivity (Sen), positive predictive value (PPV), dice similarity index (DSI), and Jaccard index (JI) of the RG-WP algorithm compared to the manual were 0.92 ± 0.04, 0.95 ± 0.04, 0.93 ± 0.02, and 0.88 ± 0.04, respectively, showing high consistency. Besides, the accuracy of the algorithm was also comparable to that of the deep learning method U-net, with Sen, PPV, DSI, and JI being 0.91 ± 0.09, 0.91 ± 0.06, 0.91 ± 0.05, and 0.91 ± 0.06, respectively.

## Introduction

Intracerebral hemorrhage (ICH) is the second most common stroke subtype, accounting for 10–15% of the stroke cases (1). The fatality rate of patients with ICH ranges from 30 to 59%, and more than 60% of the survivors have poor prognoses (2). Compared to ischemic stroke, cerebral hemorrhage is more likely to occur in younger patients and is more likely to be fatal or permanently disabling (3). Unlike ischemic stroke, there is no major breakthrough in a definitive treatment of cerebral hemorrhage. In addition to measures like optimizing blood pressure and correcting coagulation function, hematoma removal by surgery generally has favorable results. According to a review from JACC Focus Seminar, with high accuracy of the assessment of bleeding location and volume, the innovation of minimally invasive surgical technology could greatly improve early hemostasis and almost complete blood clot removal, which may lead to the next major progress in the treatment of cerebral hemorrhage (4). Moreover, since cerebral hemorrhage is associated with significant morbidity and mortality, it is crucial to assess the prognosis of patients with reasonable accuracy to facilitate decisions on initiating aggressive medical interventions or to focus on palliative care.

Computed tomography (CT) is usually the first method used in medical images due to its high efficiency, low cost, and high resolution (5). Therefore, accurate estimation of hematoma volume based on CT images is helpful in a clinic (6). Hemorrhage segmentation performed by an experienced clinician slice-by-slice has high accuracy and is usually considered as ground truth, but this method is time-consuming and laborious. On the other hand, the ABC/2 is a simple and convenient method widely used in clinical practice (7) as it can be easily implemented and as it quickly yields an estimation of hemorrhage volume. In this method, clinicians select the layer with the largest bleeding area, and the length of the longest diameter of this layer is measured and defined as A. Then, a vertical line perpendicular to the axis is drawn and its length is defined as B. The product of bleeding layers number and layer thickness is defined as C. At this point, the approximate value of bleeding volume can be estimated by the formula ABC/2 on the assumption that the hemorrhage is an ellipsoid (7). However, this method is known to have low accuracy (8, 9) and tends to overestimate the hemorrhage, especially when in face of a large hematoma area, an irregular shape, or a cerebral stem hemorrhage (10).

Therefore, numerous studies have been conducted to develop automatic or semi-automatic segmentation methods for brain CT image segmentation to assist clinical diagnosis and treatment. The thresholding-based method (11–13) and the active contour method (14–16) are two widely used approaches. Loncaric et al. (17) have made a large contribution by using Fuzzy C-Means (FCM), and then the interpretation was performed with a rule-based system. Bhadauria et al. (18) used the FCM clustering algorithm combined with the level set model to segment lesion areas and achieved great segmentation results. Chan (19) focused on small bleeding lesions, applied top-hat transformation and left-right ownership for morphological processing of CT images, and segmented hemorrhage by thresholding. Chan et al. (20) proposed a region model based on minimizing Mumford-Shah function. Patrel et al. (21) proposed a method based on multi-atlas preprocessed by label fusion and then used a geodesic active contour to refine the segmentation. However, most of these methods require a large number of samples for model training. Since it is time-consuming and involves laborious labeling of brain CT images, public data of brain CT images were rare. Hence, only small sample data could be obtained, which limited the application of these methods on CT image segmentation of cerebral hemorrhage. Besides, deep learning has made remarkable achievements in recent years (22, 23). Based on deep learning, accurate three-dimensional estimation of ICH could be carried out. However, high-throughput computing workstations and advanced graphics card were often required for this technology (24, 25), which set an obstacle to the widespread adoption of deep learning models.

As one of the classical image segmentation ideas, the region growing method has the characteristics of simplicity, rapidity, stability, and maturity (26). This study aimed to derive and validate a region growing method based on watershed preprocessing for hematoma segmentation and quantification on CT images of patients with ICH to enrich the toolbox of segmenting and quantifying the hemorrhage to make it more accurate and effective.

## Materials and Methods

### Patients and CT Images Data

The CT images in this study were retrospectively collected from patients with spontaneous ICH admitted to the neurosurgery department of a large Grade III Level hospital from January 2018 to June 2019 in China. The in-plane resolution of the CT images was 0.726 × 0.644 mm and the slice thickness was 7.2 or 9 mm. The window width and center were set to 100 hounsfied unit (HU) and 45 HU, respectively. Raw CT data was collected and converted into the DICOM (Digital Imaging and Communications in Medicine) format. This study was examined and approved by the Biomedical Ethics Review Committee of West China Hospital, Sichuan University [Approval Number: 2020 Review (716)]. Informed consent was obtained from all individual participants included in the study.

### Image Processing and Segmentation

#### Manual Segmentations

To evaluate the performance of the proposed algorithm, a series of two-dimensional (2D) slices of the patients were firstly traced by experienced clinicians manually in the open-source software ITK-SNAP version 3.8 (www.itksnap.org). Based on the labeled voxels, the volume of the hemorrhage was calculated in milliliters. The manual segmentation was performed by 2 independent raters who were blind to patient identity and clinical information. The measurements of the two raters were averaged to produce an average volume as the value for validation of the proposed method.

#### Hemorrhage Volume Estimated by ABC/2

The hemorrhage volume was also estimated by the formula ABC/2 to serve as a comparison for method evaluation. All these values were measured and marked by clinicians on ITK-SNAP software in millimeters. Then, the ICH volume was calculated based on the formula ABC/2. Like the manual segmentation, the ABC/2 was also operated independently by two raters and averaged.

### Theory of the Watershed Algorithm and the Region Growing Method

#### Watershed Algorithm

The watershed algorithm is a classic image segmentation method based on topography. It was first introduced into the field of image processing by Drivable and Lantuejoul (27). The traditional watershed algorithm is vulnerable to noise, leading to oversegmentation. This problem could be solved by the marked-based watershed algorithm. It has a strong ability to suppress noise to obtain connected and closed precise contours so as to obtain multiple connected regions. A marked-based watershed algorithm was adopted in this study. Multiple minimum points and their information could be obtained through watershed algorithm processing and then can be used as seed points for region growing.

#### Region-Growing Algorithm

The first major contribution to region growing was the early study of Muerle and Allen (28). The core idea of this method is to consider the relationships between adjacent pixels and then merge pixels with similar properties into one region. Its primary steps include (1) selecting a set of seed pixels representing the target region; (2) establishing growing criteria for pixels to be contained into the target region; and (3) establishing rules for the growth to stop.

### Segmentation Based on the Proposed RG-WP Algorithm

**Figure 1:**
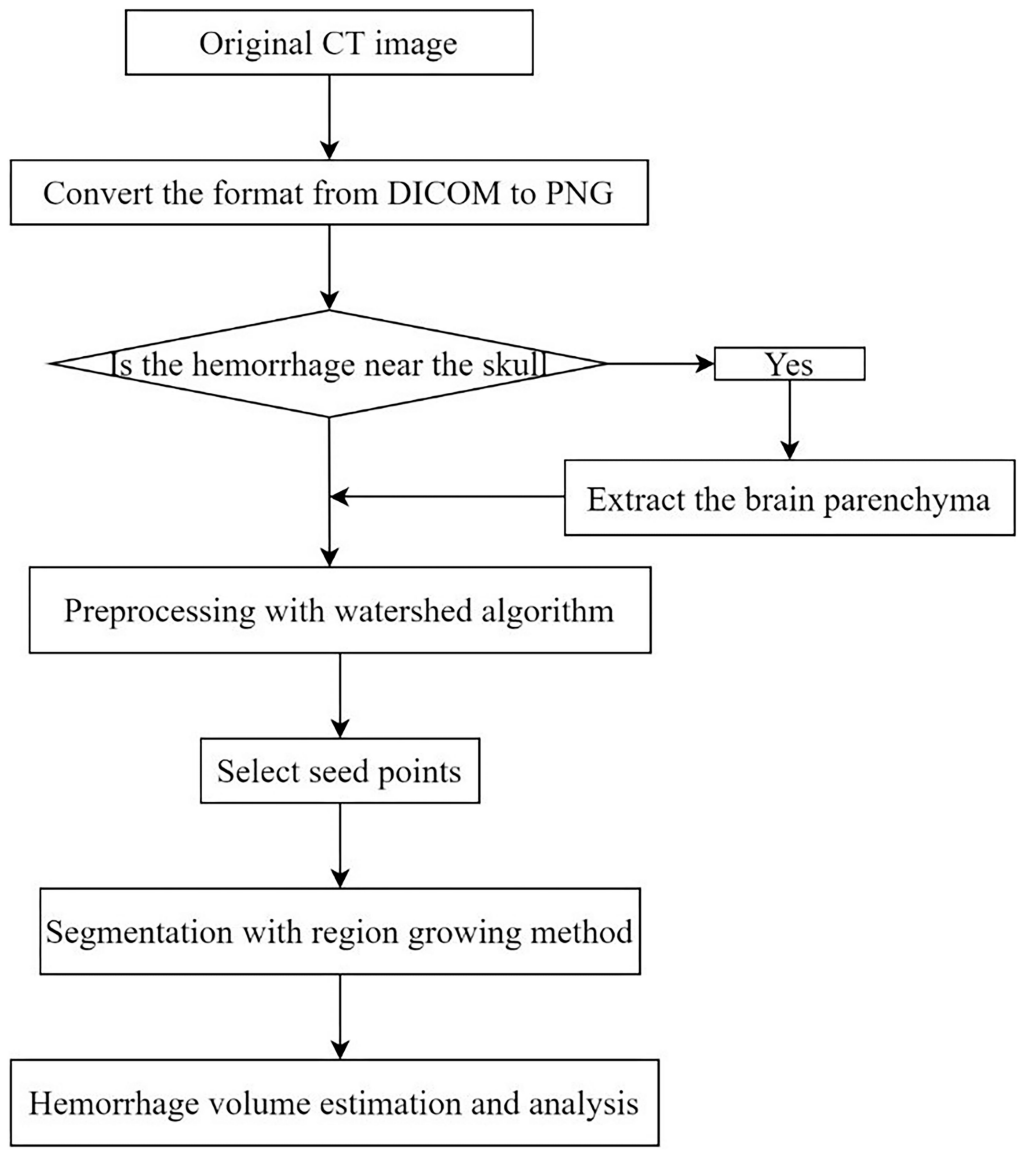
Workflow diagram of hemorrhage segmentation in this study.

Segmentation of the region growing algorithm based on watershed preprocessing (RG-WP) was mainly composed of two processes: seed point extraction based on a watershed algorithm and hemorrhage segmentation based on a region growing algorithm. When the hemorrhage was close to the skull, the brain parenchyma was firstly separated from the skull and then the hemorrhage was segmented from the brain parenchyma. The implementation details were depicted in the following sections. Figure 1 is a workflow diagram of the segmentations in this study. The proposed RG-WP algorithm was written in MATLAB (https://www.mathworks.com).

#### Seed Point Extraction by the Watershed Algorithm

**Figure 2:**
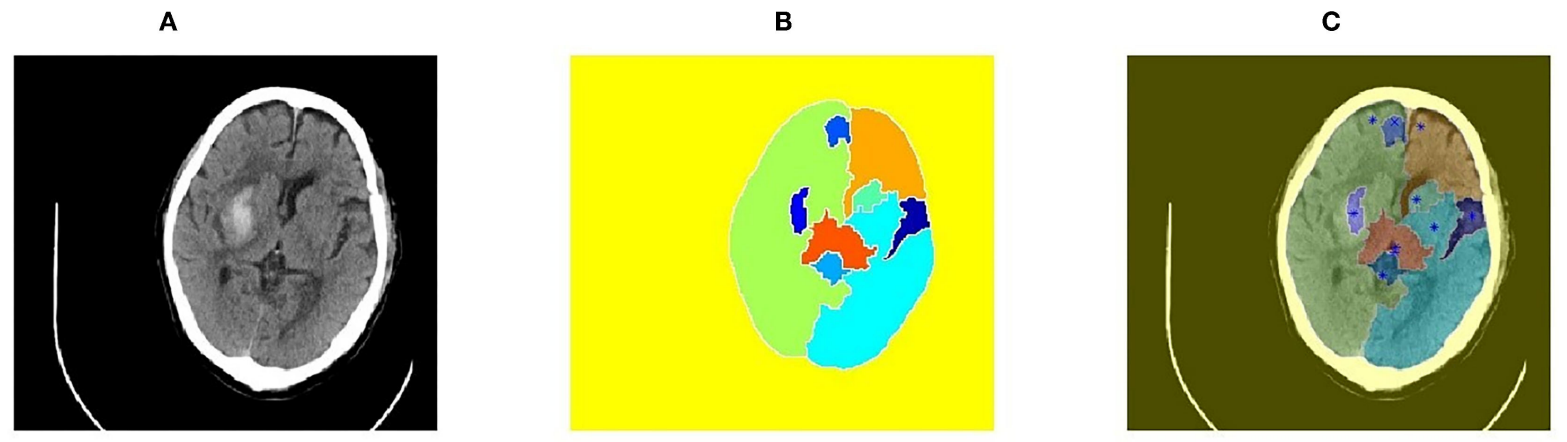
Seed point selection results based on watershed algorithm. **(A)** Original image of brain CT; **(B)** Segmentation results of marker-based watershed; **(C)** Seed points generated by watershed algorithm.

The initial seed point of the region growing method determines the direction of region growing. Selecting an optimal seed point is a key step of image segmentation. There are two ways to select seed points: automatic selection and artificial selection. Automatic selection is suitable for targeted segmentation of a specific range. For example, points with the maximum brightness could be selected as seed points in CT images. However, due to massive interference and the case of multiple hemorrhages, automatic selection is not applicable on the CT images of cerebral hemorrhage. Single artificial selection also faces large identification errors. Therefore, in this study, the watershed algorithm was proposed to initially preprocess images, and then, the seed points were further determined by manual selection. The seed point selection results are shown in Figure 2. Through the lowest points obtained by marker-based watershed algorithm, the problem that the seed points that were artificially selected may not be optimal was solved.

#### The Criterion of Region Growing

In region growing method, the growing criterion is crucial. One of its ways was based on the grayscale difference, namely the difference between the grayscale values of the seed pixels and adjacent pixels. Its expression was:


(1) $$\begin{array}{r} R=f\left(x,y\right)-f\left(i,j\right), \end{array}$$

where the (*x, y*) and (*i, j*) are the position values of the seed pixel and adjacent pixel, respectively. In this study, the grayscale range of images was firstly normalized into the range of 0 to 1. If *R* was within a certain range, the pixel to be examined was regarded as similar to the seed points. Conversely, if it exceeded the range, it was considered non-similar.

For the growing criteria, it was obtained through two steps. Firstly, the first region growing was based on a small growing threshold determined by empirical values. Then, based on the results of the first region growing, a more proper threshold value for the second region growing could be obtained by maximizing inter-class variance. The steps of the detailed process are as follows:

##### The First Region Growing

**Figure 3:**
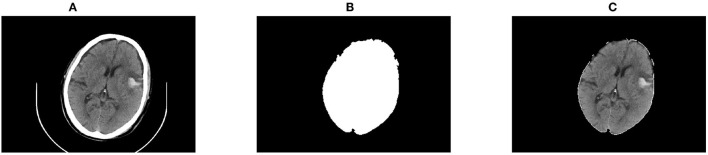
Results of the first region growing. **(A)** The original CT image; **(B)** Segmentation of the first region growing; **(C)** Restoration of the first region growing results.

The first region growing was to segment the hemorrhage with a growing threshold, artificially determined by empirical values. The difference of the growing criteria was set to 0.30, and the connected neighbors were 4 in this study. Results of the first growing are shown in Figure 3.

##### The Second Region Growing

**Figure 4:**
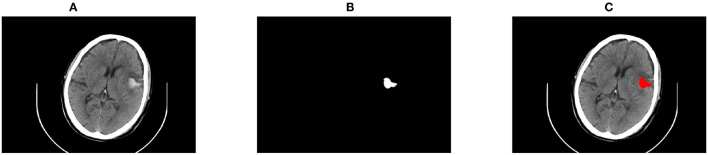
Results of the second region growing segmentation. **(A)** The original CT image; **(B)** Segmentation of the second region growing; **(C)** Restoration of the second region growing results.

Based on the region obtained by the first region growing, a more accurate growth threshold for the second region growing could be obtained by maximizing inter-class variance. The fundamental definition of the maximum was to maximize the average grayscale difference between the foreground and the background of an image. In this way, the final segmentation region could be obtained, as shown in Figure 4. The volume of the hemorrhage was computed by multiplying all the segmented pixels.

#### Deal With Hemorrhage Close to the Skull

**Figure 5:**
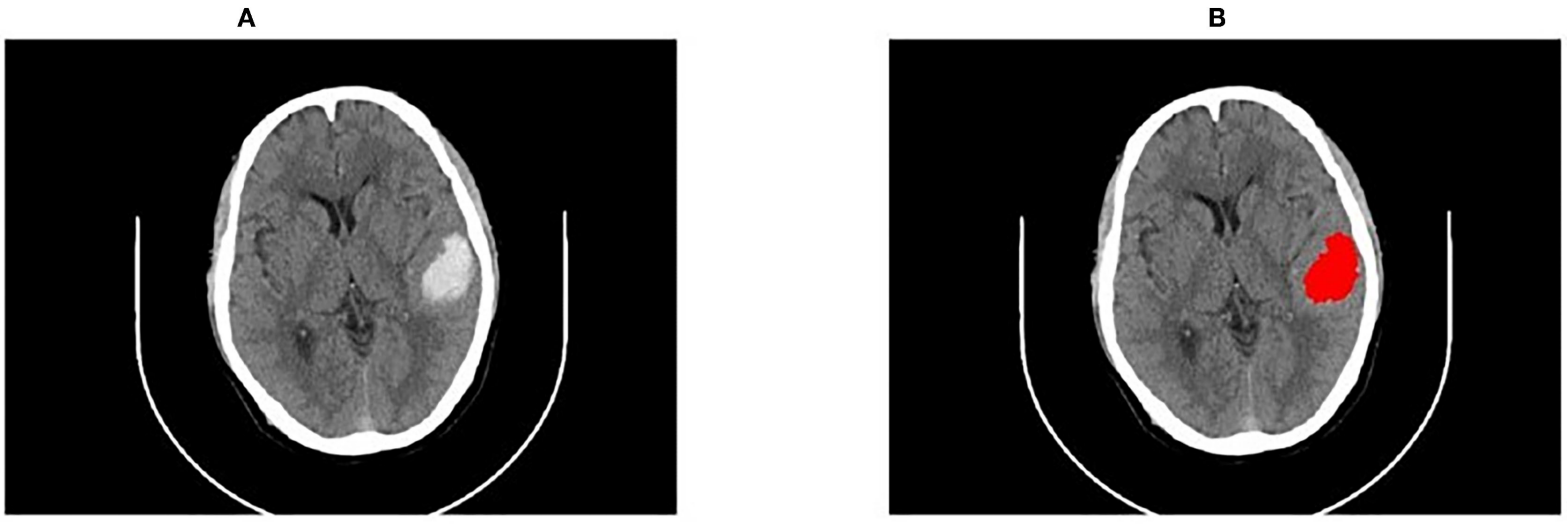
An example of segmentation close to the skull. **(A)** The original CT image; **(B)** Restoration of the segmentation results.

When ICH was close to the skull, it was liable to be connected to the head, bringing bias to the identification of hemorrhage. To this end, this study first extracted the brain parenchyma with the region growing method and then the hemorrhage was segmented from the parenchyma. An example of segmentation close to the skull is shown in Figure 5.

### Evaluation Methodology

To confirm the performance of the proposed RG-WP algorithm, the sensitivity (Sen), the positive predictive value (PPV), the dice similarity index (DSI) (29), and the Jaccard index (JI) of the algorithm compared to manual segmentation were calculated. The formulas were as follows:


(2) $$\begin{array}{r} \text{Sen}=\frac{TP}{TP+FN}, \end{array}$$

(3) $$\begin{array}{r} \text{SPPV}=\frac{TP}{TP+FP}, \end{array}$$

(4) $$\begin{array}{r} \text{SDSI}=\frac{2TP}{2TP+FP+FN}, \end{array}$$

(5) $$\begin{array}{r} \text{SJI}=\frac{\left|AV\cap GT\right|}{\left|AV\cup GT\right|}, \end{array}$$

where AV represented the region divided by the algorithm and GT represented the region divided manually. When a manually segmented hematoma was also correctly identified by the algorithm as hemorrhage, it was called true positive (TP) and false negative (FN) when it was incorrectly identified as non-hematoma. True negative (TN) was defined when a manually marked area that was correctly identified by the algorithm as non-hematoma, and false positive (FP) was defined when it was incorrectly identified by the algorithm as hematoma. Meanwhile to make a comparison of the RG-WP algorithm with the widely accepted deep learning method U-net (30, 31), the same CT image data set was also segmented by the U-net, and the similarity metrics (Sen, PPV, DSI, JI) were also calculated.

### Statistical Analysis

The hemorrhage volumes were first presented with the range, mean, median, and IQR. Bland–Altman plots were drawn to assess the agreement between methods (32). The consistency of different methods was further assessed by ICC, and similarity metrics (Sen, PPV, DSI, and JI) were also calculated. Differences among methods were tested with the Friedman test followed by the Dunn's multiple comparison test. For manual segmentations by two independent raters, to avoid the probable bias caused by subjectivity or interrater variability in the process of manual segmentation, the paired *t*-test was performed to confirm statistical validity. Reliability between the two raters was demonstrated by the ICC and the Bland–Altman plots. All statistical analyses were conducted with R version 4.0.3, and *P* < 0.05 was considered significant.

## Results

### Inter-rater Reliability

The mean volume of the manual segmentations by the two independent raters were 9.74 ± 7.73 and 9.64 ± 7.79 (Table 1). Volume values obtained by the two raters were not statistically different (*t* = 1.731, *P* = 0.09). The ICC of manual segmentations by the two raters were 0.998 (95% *CI*: 0.997–0.999) and of volumes estimated *via* ABC/2 by the two raters were 0.996 (95% *CI*: 0.993–0.998). Interrater agreement by manual segmentation and by ABC/2 was also depicted by the Bland-Altman plots seen in Supplementary Figure 1. The high intraclass correlation and small volume deviations indicated promising interrater agreement for the manually acquired data.

**Table 1:** Summary of segmentations manually by two independent raters.

	**Volumes of hemorrhage (mL)**		**Times of segmentation (s)**	
	**Rater A**	**Rater B**	**Rater A**	**Rater B**
Min	0.66	0.43	540	515
Max	29.44	29.10	900	850
μ ±σ	9.74 ±	9.64 ±	720.40 ±	684.32 ±
	7.73	7.79	180.55	150.21

### Agreement Analysis Among the RG-WP Algorithm, Manual Segmentation, and ABC/2

A highly strong agreement between volumes obtained by the RG-WP algorithm and manual segmentation was illustrated by an ICC of 0.998 (95% *CI*: 0.997–0.999). The agreement between ABC/2 and the manual segmentation was also strong with an ICC of 0.972 (95% *CI*: 0.953–0.984). The mean deviation of volumes by the algorithm from manual segmentation was −0.12 ml (range: −1.05–1.16), while that of the ABC/2 from manual segmentation was 1.05 ml (range: −0.77–9.57) (Table 2). The deviation of ABC/2 from manual segmentation was larger than that of the algorithm from the manual. No statistically significant differences were found among the three approaches (*P* > 0.05).

**Table 2:** Segmentations comparison among the RG-WP algorithm, manual, and ABC/2.

	**RG-WP algorithm vs. manual**	**ABC/2 vs. manual**	**ABC/2 vs. RG-WP algorithm**
**Deviation of volumes, mL**			
Range (min, max)	(−1.05, 1.16)	(−0.77, 9.57)	(−0.86, 9.37)
Mean	−0.12	1.05	1.18
Median	−0.09	0.44	0.49
IQR	−0.31, 0.04	0.13, 1.41	0.11, 1.66
95% CI	−1.04, 0.80	−2.28, 4.39	−2.37, 4.72
ICC (95% CI)	0.998 (0.997, 0.999)	0.972 (0.953, 0.984)	0.968 (0.945, 0.981)

**Figure 6:**
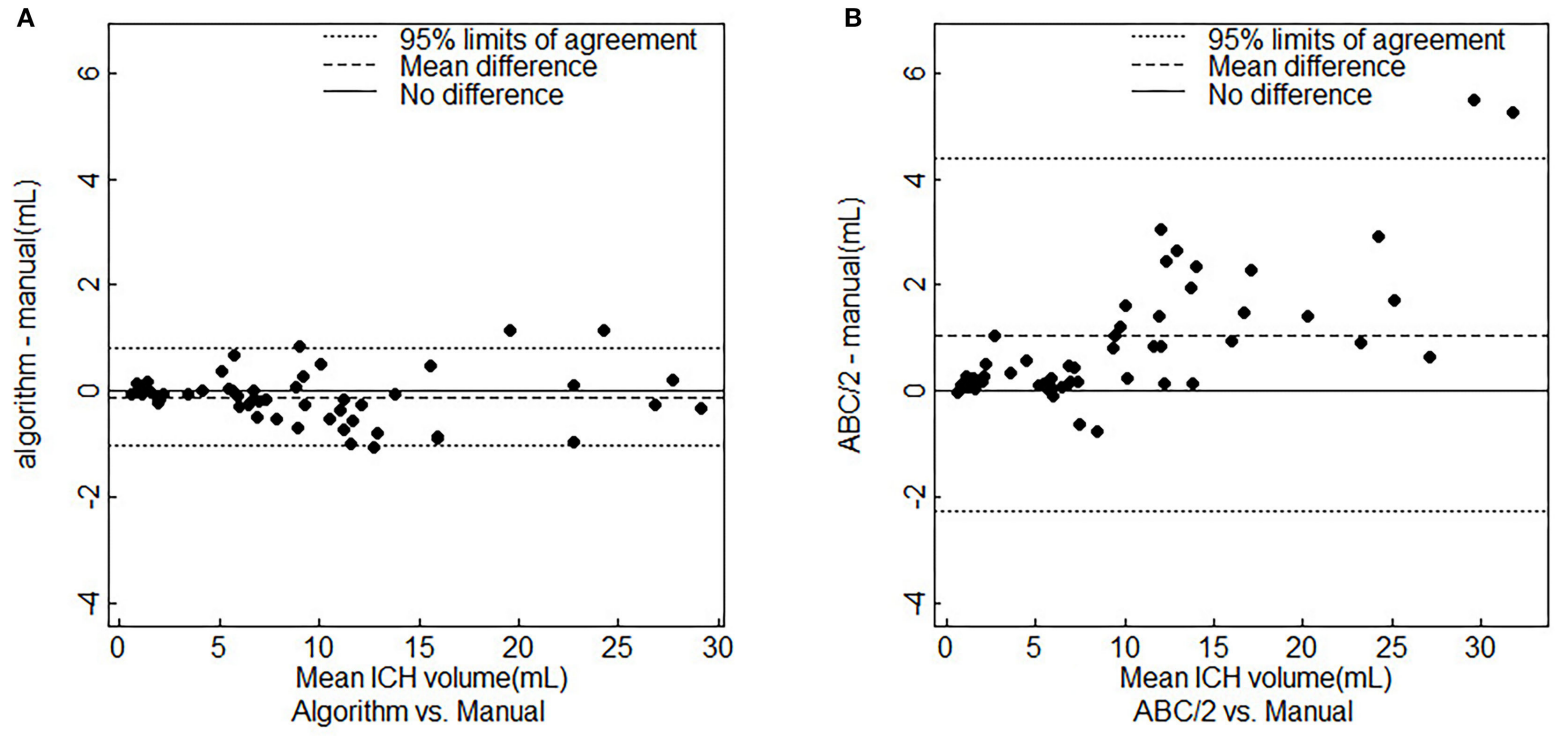
Bland-Altman plots for agreement analysis. **(A)** RG-WP algorithm vs. manual segmentations. **(B)** ABC/2 vs. manual segmentations. ICH, intracerebral hemorrhage.

Figures 6A,B showed the Bland–Altman plots of the RG-WP algorithm and ABC/2 compared with the manual segmentation, respectively. It seemed that ABC/2 tended to overestimate the hemorrhage. The volume deviation scale of ABC/2 was larger than that of the algorithm. Besides, it could be seen from Figure 6B that the accuracy of ABC/2 could not be guaranteed, especially when the hemorrhage was large. When the hemorrhage volume was larger than 10 ml, the deviation of ABC/2 overestimation was large, up to 9.37 mL, while the deviation of the RG-WP algorithm was always smaller and more stable.

As for the segmentation efficiency, the processing time by the algorithm was 18 s on average per patient while the time was 702.36 s, ~12 min by the manual segmentation. The time was reduced by 684.36 s per patient by the algorithm compared with manual segmentation.

### Comparison of the RG-WP Algorithm and the U-Net

**Figure 7:**
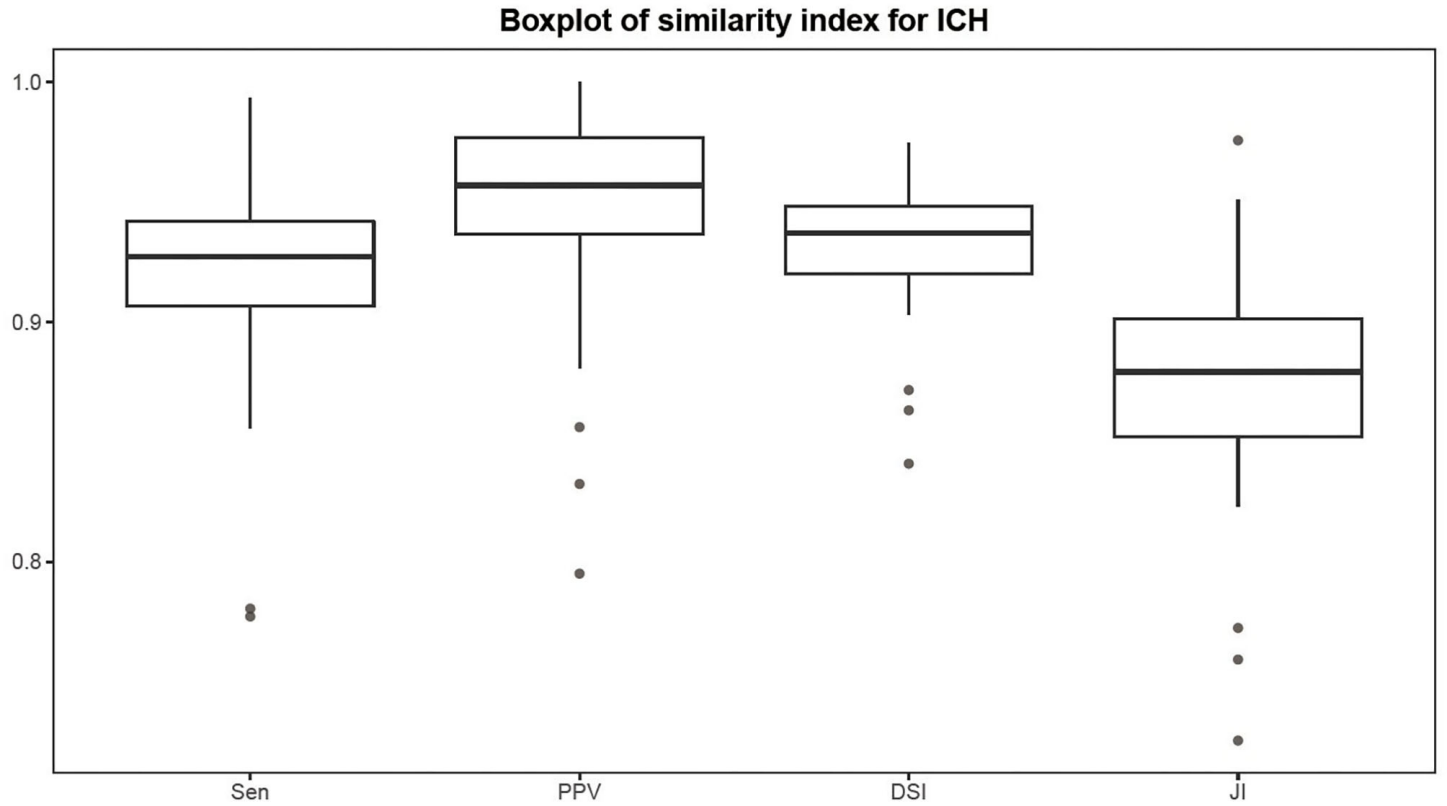
Boxplot of similarity metrics.

**Figure 8:**
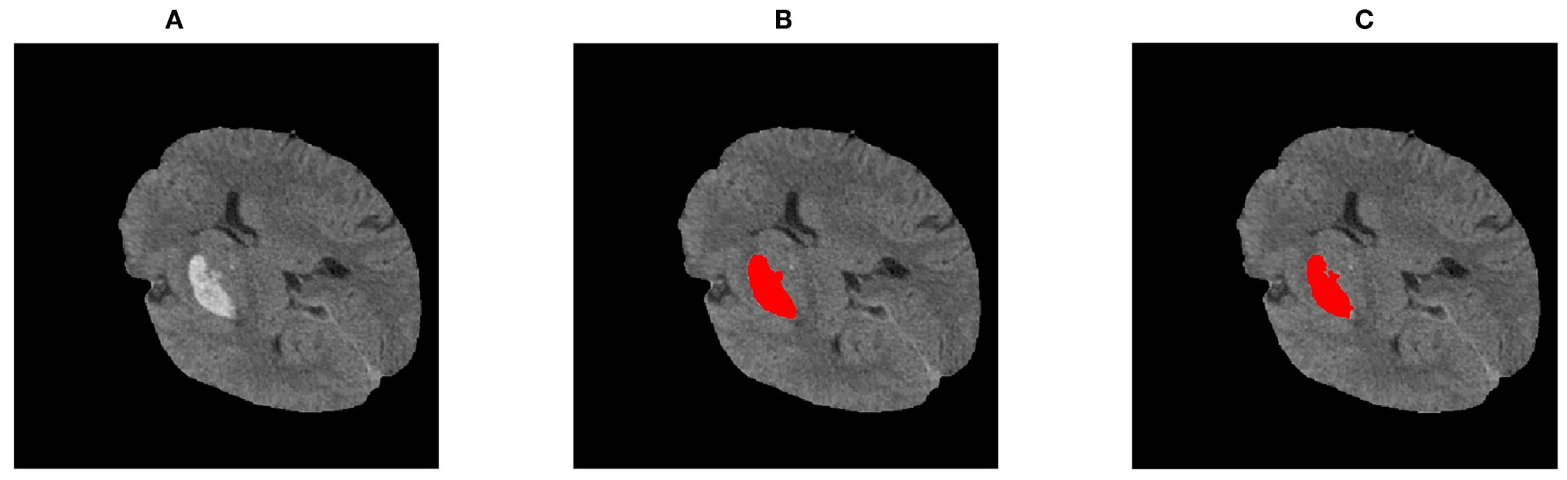
Segmentations by different approaches: **(A)** the original image; **(B)** segmentation by standardized U-net; **(C)** segmentation by the RG-WP algorithm.

The Sen, PPV, DSI, and JI of the RG-WP algorithm compared with the manual were 0.92 ± 0.04, 0.95 ± 0.04, 0.93 ± 0.02, and 0.88 ± 0.04, respectively. Distributions of the metrics are presented by a boxplot in Figure 7. The medians of the four similarity metrics were all above 0.85, indicating strong consistency of the algorithm and manual segmentation. For U-net, the four metrics were 0.91 ± 0.09, 0.91 ± 0.06, 0.91 ± 0.05, and 0.91 ± 0.06, respectively. The metrics of the proposed algorithm were comparable to the U-net. Figure 8 was an example of segmentation by the RG-WP algorithm and U-net.

## Discussion

This study proposed and validated a region growing method based on watershed preprocessing to improve the efficiency and accuracy of the segmentation and quantification for spontaneous ICH on CT images. When compared to manual segmentation, the proposed RG-WP algorithm showed promising accuracy. At the same time, its accuracy was not only superior to the ABC/2 but also comparable to the deep learning method U-net. Meanwhile, segmentation by the RG-WP algorithm was computationally efficient, requiring only 18 s to segment and measure volumes per patient, while it took ~12 min for a clinician to manually segment the hemorrhage per patient.

One major target of this study was to improve the accuracy of hemorrhage segmentation and quantification. In the clinic, ABC/2 was currently widely used for ICH quantification, but it was known to have low accuracy in face of large or irregularly shaped hematomas (8, 9). The results of this study confirmed that ABC/2 tended to overestimate the hemorrhage, especially when the hemorrhage was large (Figure 6). On the contrary, the RG-WP algorithm showed higher accuracy and stronger stability when dealing with diverse images. Its segmentation volume deviation and variation range were both less than those of the ABC/2, even when dealing with large hemorrhages (Table 2). The ICC of ABC/2 compared to manual segmentation was lower than that of the RG-WP algorithm compared to the manual, indicating a higher accuracy of the algorithm than ABC/2. In addition to the simple geometric estimation method by ABC/2, previous studies had applied methods like deep learning for medical image segmentation and have achieved good results. Among them, the U-net was a widely proven effective method (30, 31, 33). Therefore, this study also compared the RG-WP algorithm with U-net. It turned out that the proposed algorithm was non-inferior to the U-net in terms of the consistency metrics compared to the manual segmentation. Furthermore, this result was confirmed by evidence from previous studies applying deep learning methods to hemorrhage segmentation (34–36). For example, in the research by Scherer et al. (34), a random forest method based on first- and second-order statistics, texture, and threshold characteristics was trained to segment the hemorrhage. It reported a mean ICC of 0.95, which was slightly lower than that of our proposed RG-WP algorithm (ICC = 0.998). Besides, the mean DSI reported in this study was 0.92, which was larger than that of Ironside et al. (35) with convolutional neural networks (CNN) (DSI = 0.894). Meanwhile, the results of the proposed algorithm were also comparable to those reported by Nag et al. (36) using the active contour Chan–Vese model (Sen = 0.71 ± 0.12, PPV = 0.73 ± 0.18, JI = 0.55 ± 0.14, DSI = 0.70 ± 0.12). Hence, both the statistical and the empirical evidence indicated that the RG-WP algorithm was non-inferior to the deep learning method in the accuracy of hemorrhage segmentation and quantification.

From an efficiency point of view, the proposed RG-WP algorithm had greatly reduced the time of image processing compared with manual segmentation. Clinically, the situation of cerebral hemorrhage is often urgent and the treatment of cerebral hemorrhage is a race against time (4). The shortening of the processing time is of great significance for reducing mortality and improving prognosis in patients with cerebral hemorrhage.

Although we have already compared the RG-WP algorithm with deep learning methods in terms of accuracy, such comparison was not thorough and fair if the operability was excluded from consideration. Specifically, the operability of a method is referred to its demand on external resources (i.e., equipment and data) to finish the desired task. The deep learning methods usually have a great demand for high-throughput equipment (24, 25), which make them difficult to be applied to underdeveloped rural areas. In contrast, the RG-WP algorithm of this study has a relatively lower demand for equipment and is more conducive to promotion in some underdeveloped primary medical institutions to help improve their medical capabilities. Furthermore, since the RG-WP algorithm does not need labeled data for supervised training, it is favorable in dealing with the commonly encountered data shortage in practice.

It should be acknowledged that this study still had limitations. From the epidemiological point of view, the sample size was relatively small and all the images were collected from one hospital, which may offer a caveat on the generalization ability of the proposed method. Although generalization ability was out of the scope of this study, such caveat deserved sufficient attention when one was trying to apply the RG-WP algorithm to other areas. Therefore, it was highly recommended that future studies consider incorporating data from multicenter to verify its generalization to different conditions.

In conclusion, this study demonstrated a favorable performance of segmentation of ICH on CT images by a region growing algorithm based on watershed preprocessing (RG-WP), which showed strong consistency with the present ground truth, manual segmentation. It was superior to the widely used ABC/2 method and non-inferior to the excellent deep learning method. Considering its fast processing speed, low requirements for equipment, and lack of the need for labeled images as a training set, it was highly expected that this method could contribute to a fast and accurate diagnosis and decision-making of patients with ICH in clinical practice.

## Data Availability Statement

The raw data supporting the conclusions of this article will be made available by the authors, without undue reservation.

## Ethics Statement

The studies involving human participants were reviewed and approved by the Biomedical Ethics Review Committee of West China Hospital, Sichuan University. The patients/participants provided their written informed consent to participate in this study.

## Author Contributions

ZZ, HW, HZ, and TZ contributed to conception and design of the study. ZZ, HW, HZ, XC, XW, SL, and TZ assembled the dataset, performed the experiments, and performed the statistical analysis. ZZ and HW wrote the manuscript. All authors contributed to the article and approved the submitted version.

## Funding

This research work was funded by the Sichuan Science and Technology Program (2020YFS0091, 2020YFS0015, 2021YFS0001-LH, and 2019-YF05-00333-SN), the Health Commission of Sichuan Province (20PJ092), the National Natural Science Foundation of China (81602935), the Chongqing Science and Technology Program (cstc2020jscx-cylhX0003), the Chengdu Science and Technology Program (2021-YF05-01585-SN), the Sichuan University (2018hhf-26 and 2018HXFH010), the Central Government Funding Items (2021zc02), and the Liangshan Prefecture Center for Disease Control and Prevention (H210322). The funders played no role in the design of the study and collection, in the analysis and interpretation of data, and in writing the manuscript.

## Conflict of Interest

The authors declare that the research was conducted in the absence of any commercial or financial relationships that could be construed as a potential conflict of interest.

## Publisher's Note

All claims expressed in this article are solely those of the authors and do not necessarily represent those of their affiliated organizations, or those of the publisher, the editors and the reviewers. Any product that may be evaluated in this article, or claim that may be made by its manufacturer, is not guaranteed or endorsed by the publisher.
