# Supplementary material for: Segmentation of Spontaneous Intracerebral Hemorrhage on CT With a Region Growing Method Based on Watershed Preprocessing
Source: Front Neurol. 2022 Mar 29;13:865023. doi: 10.3389/fneur.2022.865023 (PMC9002175; doi:10.3389/fneur.2022.865023)
Supplement: Supplementary file 1 [file Data_Sheet_1.PDF]

Supplementary Material

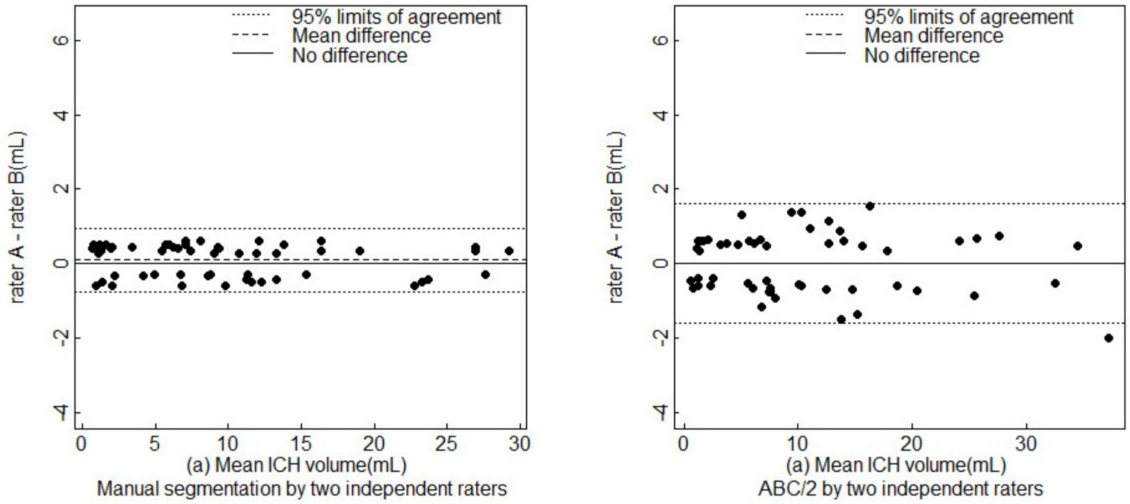

Supplementary Figure 1 Interrater agreement in manual measurements. (a)Manual segmentation by two independent raters. (b) ABC/2 by two independent raters
